# Proteomic analysis reflects an environmental alkalinization-coupled pH-dependent mechanism of regulating lignocellulases in *Trichoderma guizhouense* NJAU4742

**DOI:** 10.1186/s13068-020-1651-0

**Published:** 2020-01-11

**Authors:** Youzhi Miao, Xing Chen, Tuo Li, Han Zhu, Siyu Tang, Dongyang Liu, Qirong Shen

**Affiliations:** Jiangsu Provincial Key Lab for Organic Solid Waste Utilization, National Engineering Research Center for Organic-based Fertilizers, Jiangsu Collaborative Innovation Center for Solid Organic Waste Resource Utilization, College of Resources and Environmental Science, Nanjing Agricultural University, Nanjing, 210095 People’s Republic of China

**Keywords:** Plant biomass, *Trichoderma*, pH, Lignocellulases, Environmental alkalinization

## Abstract

**Background:**

Filamentous fungi have the ability to efficiently decompose plant biomass, and thus are widely used in the biofuel and bioprocess industries. In process, ambient pH has been reported to strongly affect the performance of the applied functional filamentous fungi. In this study, *Trichoderma guizhouense* NJAU4742 was investigated under the fermentation of rice straw at different initial pH values for a detailed study.

**Results:**

The results showed that NJAU4742 strain could tolerate ambient pH values ranging from 3.0 to 9.0, but had significantly higher growth speed and extracellular enzyme activities under acidic conditions. At low ambient pH (< 4), NJAU4742 strain achieved rapid degradation of rice straw by elevating the ambient pH to an optimal range through environmental alkalinization. Further proteomic analysis identified a total of 1139 intracellular and extracellular proteins during the solid-state fermentation processes, including the quantified 190 carbohydrate-active enzymes (CAZymes) responsible for rice straw degradation, such as 19 cellulases, 47 hemicellulases and 11 chitinases. Meanwhile, the analysis results clearly showed that the secreted lignocellulases had a synergistic trend in distribution according to the ambient pH, and thus led to a pH-dependent classification of lignocellulases in *T. guizhouense* NJAU4742.

**Conclusions:**

Most functional lignocellulases were found to be differently regulated by the ambient pH in *T. guizhouense* NJAU4742, which had the ability of speeding up biomass degradation by elevating the ambient pH through environmental alkalinization. These findings contribute to the theoretical basis for the biodegradation of plant biomass by filamentous fungi in the biofuel and bioprocess industries.

## Background

Plant biomass, as the most abundant natural material on earth, mainly consists of cellulose (40–50%), hemicellulose (25–30%) and lignin (15–20%) [1]. For a long time, scientists have tried their best to overcome the structural recalcitrance and time-consuming degradation process of plant biomass [2–4]. Detailed in structure, cellulose and hemicellulose are the most abundant natural polysaccharides, where the former exists mostly as a crystalline structure [5, 6], and the latter exists as a mixture of xylan, glucomannan, arabinogalactan, xyloglucan and galactoglucomannan [7]. Hemicellulose is usually decorated with various side-chain substituents, such as acetyl, α-arabinosyl, α-glucuronyl and 4-*O*-methylglucuronycyl residues [7]. As a complex phenolic polymer, lignin is composed of three monolignol monomers that can be methoxylated to various degrees: *p*-coumaryl alcohol, coniferyl alcohol, and sinapyl alcohol. These lignols are incorporated into lignin in the form of the phenylpropanoid *p*-hydroxyphenyl (H), guaiacyl (G), and syringyl (S) units, respectively [8]. Finally, by physical and chemical forces, all three different polymers form a 3D microstructure in the plant cell wall, which is very stable and shows strong resistance to hydration [9].

So far, plant biomass is widely used in two areas: biofuel and bioorganic fertilizer [10], in which filamentous fungi are employed as the plant biomass-degraders for their highly efficient secretion of many synergistic enzymes, such as cellulases, hemicellulases, pectinases and ligninases. However, it is still a challenge to completely degrade plant biomass in an economical amount of time just with the secreted enzymes from filamentous fungi. In the biofuel industry, acid or alkaline pretreatment is used and will partly destroy and significantly loosen the entire stable structure of plant biomass, thus providing a less recalcitrant and much more accessible material for the hydrolytic enzymes [11]. Similarly, in the field of bioorganic fertilizer, amino acids with animal origins are added into plant biomass for highly efficient fermentation by functional filamentous fungi, aiming to supply nutrients, slightly pretreat and prevent the growth of other microbes [12]. Certainly, all of these treatments would significantly change the subsequent environmental pH of the next step regardless of biofuel or bioorganic fertilizer production. Additionally, biological organic fertilizer containing the functional filamentous fungi would be applied to different farmlands, which have fluctuant natural pH values ranging from 4.0 to 9.0 [13]. These facts drew our attention to investigate the effect of pH on the growth of filamentous fungi, or more specifically, focus on the effect of pH on the regulation of lignocellulases in functional filamentous fungi.

pH regulation of lignocellulases in filamentous fungi has already been investigated previously. For example, in *Aspergillus nidulans*, XlnB (xylanase) and AbfB (α-l-arabinofuranosidase) were acid-expressed, while XlnA (xylanase) was alkaline-expressed [14]. In *Botrytis cinerea*, protein gel identification showed that three hydrolases (α-1,2-mannosidase, α-l-arabinofuranosidase and β-1,3-glucosidase) were up or specifically accumulated at pH 4.0, while four other hydrolases (exocellulase, arabinogalactan endo-1,4-β-galactosidase, rhamnogalacturonan acetyl esterase and exoarabinanase) were up or specifically accumulated at pH 6.0 [15]. Transcriptome analysis of *Trichoderma reesei* also indicated that a series of genes encoding cellulases and hemicellulases were selectively induced under various pH values [16]. Furthermore, deeper studies revealed that a pH regulatory system could be mediated by the transcription factor of PacC, which was responsible for differential protein expression at different pH values [14, 17]. It was reported that knocking out the *pacC* gene could significantly affect both cellulase and xylanase activities in two model filamentous fungi, *Trichoderma reesei* and *Neurospora crassa* [18, 19]. These previous results showed that a PacC-mediated mechanism likely exists as a general way of regulating lignocellulases in filamentous fungi. However, only portions of enzymes or sporadic indirect pieces of evidence (transcriptome sequencing) were provided in those previous studies, and very few studies have tried to entirely investigate the change in all proteins in response to pH in a filamentous fungus.

*Trichoderma guizhouense* NJAU4742 strain, which was isolated from compost, was widely used as a functional active component of biological organic fertilizer. In this study, NJAU4742 strain was fermented at different initial pH values to investigate the effects on degradation of the sole carbon source (rice straw). In the whole process, the growth conditions and extracellular lignocellulose activities were analyzed in detail. Simultaneously, the proteomic analysis using the Sequential Window Acquisition of all Theoretical fragment ions (SWATH) technique provided a specific description of how pH affected the intracellular and extracellular proteins synthesized by NJAU4742 strain.

## Results

### Pre-evaluating the ability of *T. guizhouense* NJAU4742 to degrade rice straw at different pH values in the submerged fermentation

**Fig. 1:**
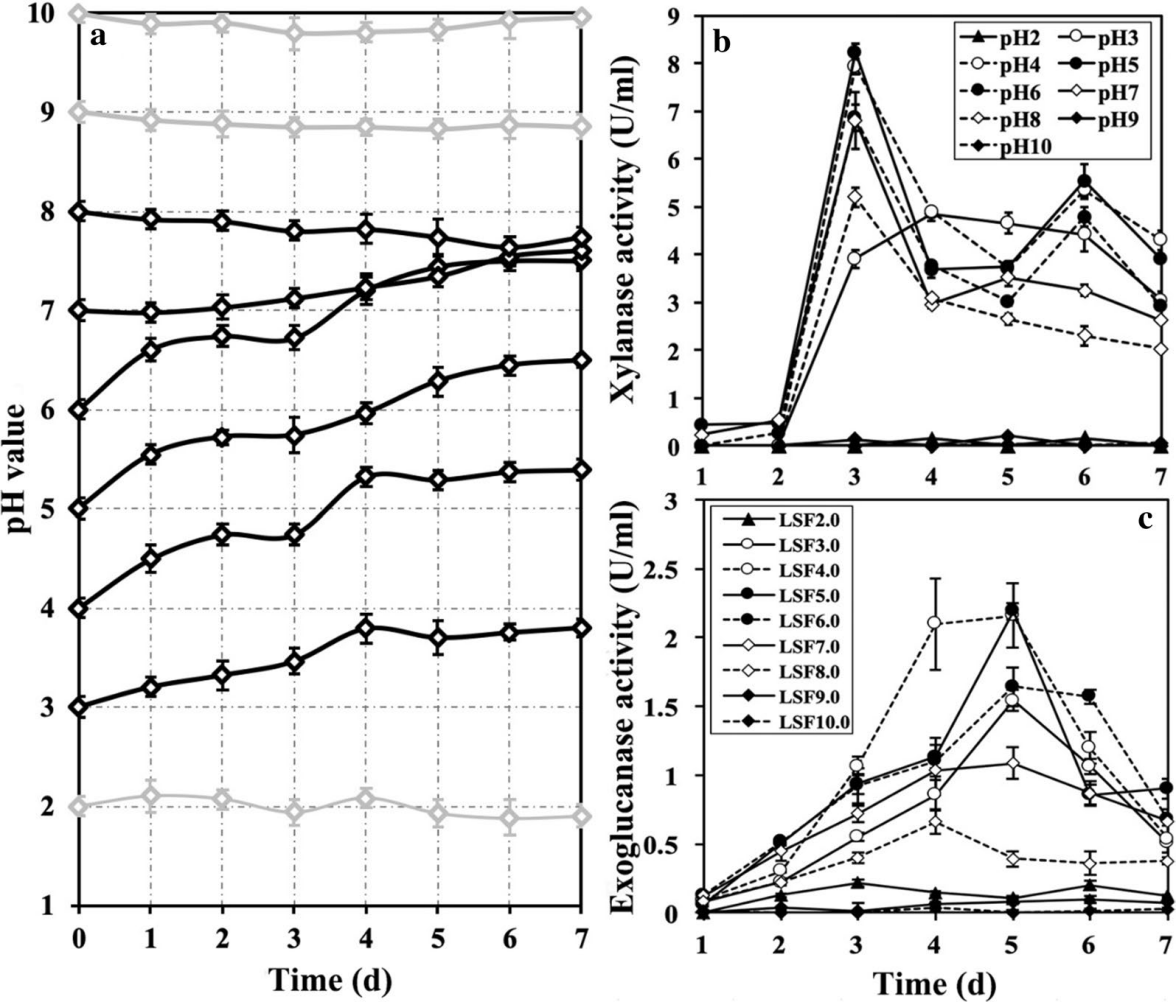
The time course of pH changes and enzyme activities under the submerged fermentation in different treatments. The submerged fermentations of rice straw by NJAU4742 strain were designed to be performed at different initial pH values (2.0, 3.0, 4.0, 5.0, 6.0, 7.0, 8.0, and 9.0). At 7 days, the environmental pH (**a**), extracellular xylanase activities (**b**) and extracellular exoglucanase activities (**c**) were detected. The bars indicate the standard error of four replicates

In this study, spores of NJAU4742 strain (1 × 10^7^/L) were inoculated into triangular flasks containing liquid minimal medium (MM) with a pH ranging from 2.0 to 10.0 and 2% (w/v) rice straw as the sole carbon source. During cultivation at 28 °C and 150 rpm, pH changes were determined, and results are shown in Fig. 1a. The growth of NJAU4742 strain could significantly regulate the ambient pH; for example, initial pH values of 3.0, 4.0, 5.0, 6.0 and 7.0 were all increased by an average increment of approximately one pH value after the cultivation of NJAU4742 strain for 7 days, but the initial pH values of 2.0, 9.0 and 10.0 remained stable throughout the entire cultivation period. Meanwhile, endoglucanase and xylanase activities were also evaluated for cellulose and hemicellulose degradation abilities, respectively, during the biodegradation process (Fig. 1b, c). Consistent with the pH changes, no enzyme activities were detected in the cultivations with the initial pH values of 2.0, 9.0 and 10.0. Surely, both could be explained by the fact that the initial high concentration of H^+^ or OH^−^ could actually inhibit spore germination and further growth of NJAU4742 strain in the submerged fermentation (data not shown). In addition, both the highest xylanase (8.23 ± 0.20 U/mL) and exoglucanase (2.19 ± 0.06 U/mL) activities were obtained in the treatment with initial pH values of 5.0 in the third and 5th day, respectively. In general, enzyme activities, in fermentations with the initial pH values ranging from 3.0 to 7.0, had no statistical differences between each other (*p* > 0.05), and were all kept in a relatively high level from the 3rd day (the average xylanase activities: 3.84–5.02 U/mL, and the average exoglucanase activities: 0.87–1.41 U/mL). All these results indicated that *T. guizhouense* NJAU4742 strain preferred acidic environment, and the ambient pH was one of the critical parameters for the lignocellulose utilization by this strain.

### The performance of NJAU4742 strain under solid-state fermentations of rice straw at different pH values

Solid-state fermentation is an important method for cultivating filamentous fungi when used in agriculture or industry. Based on the data obtained from the submerged fermentations, pH values of 2.0, 3.0, 6.0, 8.0 and 9.0 were chosen as the initial ambient pH values for the solid-state fermentations of NJAU4742 strain using rice straw as the sole carbon source. During the fermentation course of 7 days, the spores germinated and grew until large amounts of green mature spores were produced (a complete growth cycle, Additional file 1: Figure S1). In solid-state fermentation, an initial pH of 6.0 could significantly accelerate the growth of NJAU4742 strain compared to other pH values, and initial pH values of 3.0 and 8.0 could also normally incubate this strain, but with some delay in the growth when compared to the former treatment. Similar to the submerged fermentation, an initial pH of 2.0 completely inhibited the spore germination of NJAU4742 strain, and a relatively small amount of mycelia were observed in the treatment with the initial pH value of 9.0.

**Fig. 2:**
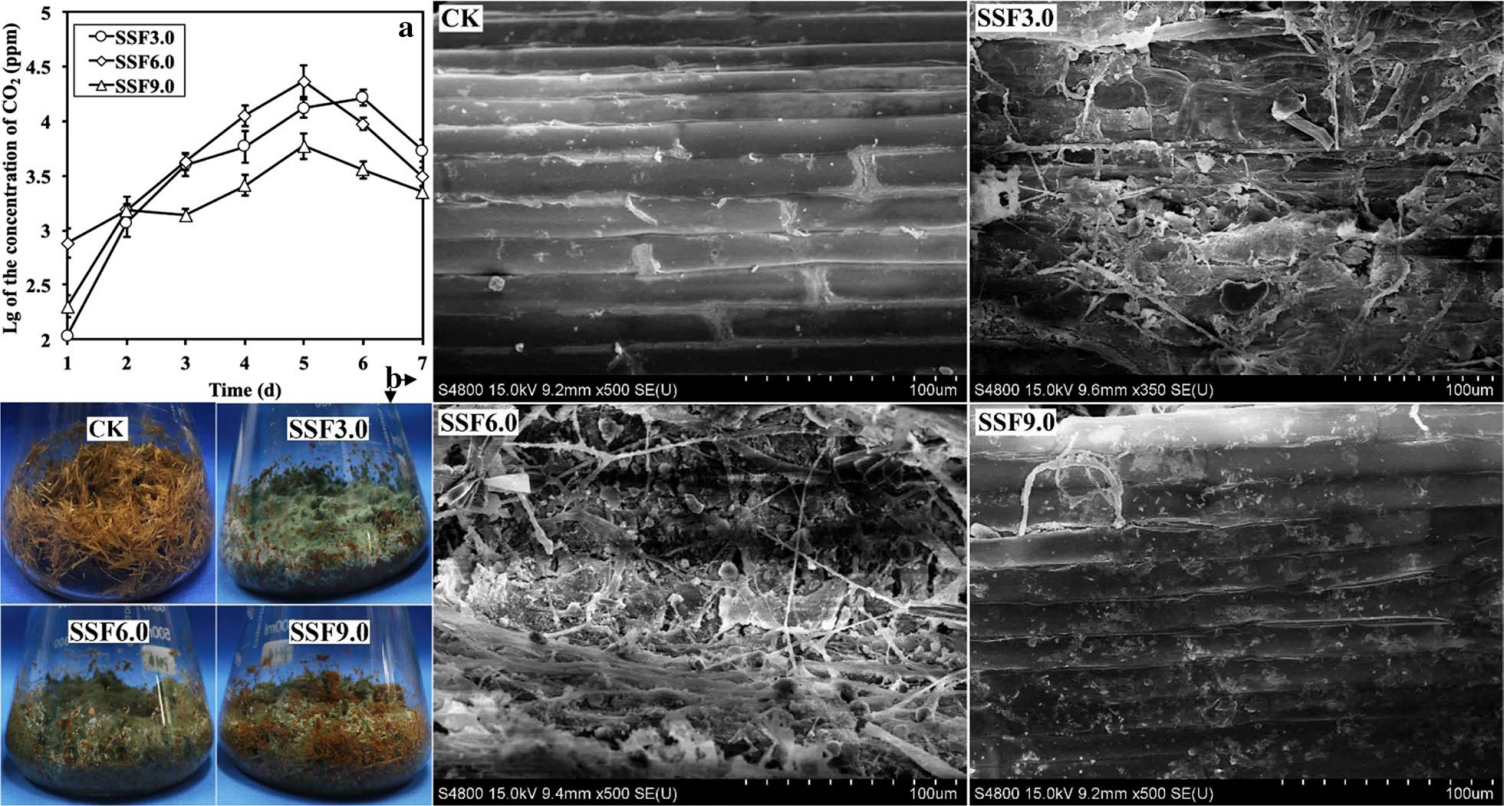
Solid-state fermentations of rice straw by NJAU4742 strain under the different treatments of SSF3.0, SSF6.0 and SSF9.0. The release of CO_2_ was detected in 7 days of solid-state fermentation (**a**). The pictures of solid-state fermentation of rice straw by NJAU4742 strain for the 7th day at pH 3.0, 6.0 and 9.0 and the scanning electron micrographs of natural rice straw (CK) and rice straw in SSF for the 7th day are shown (**b**). The bars indicate the standard error of three replicates

**Fig. 3:**
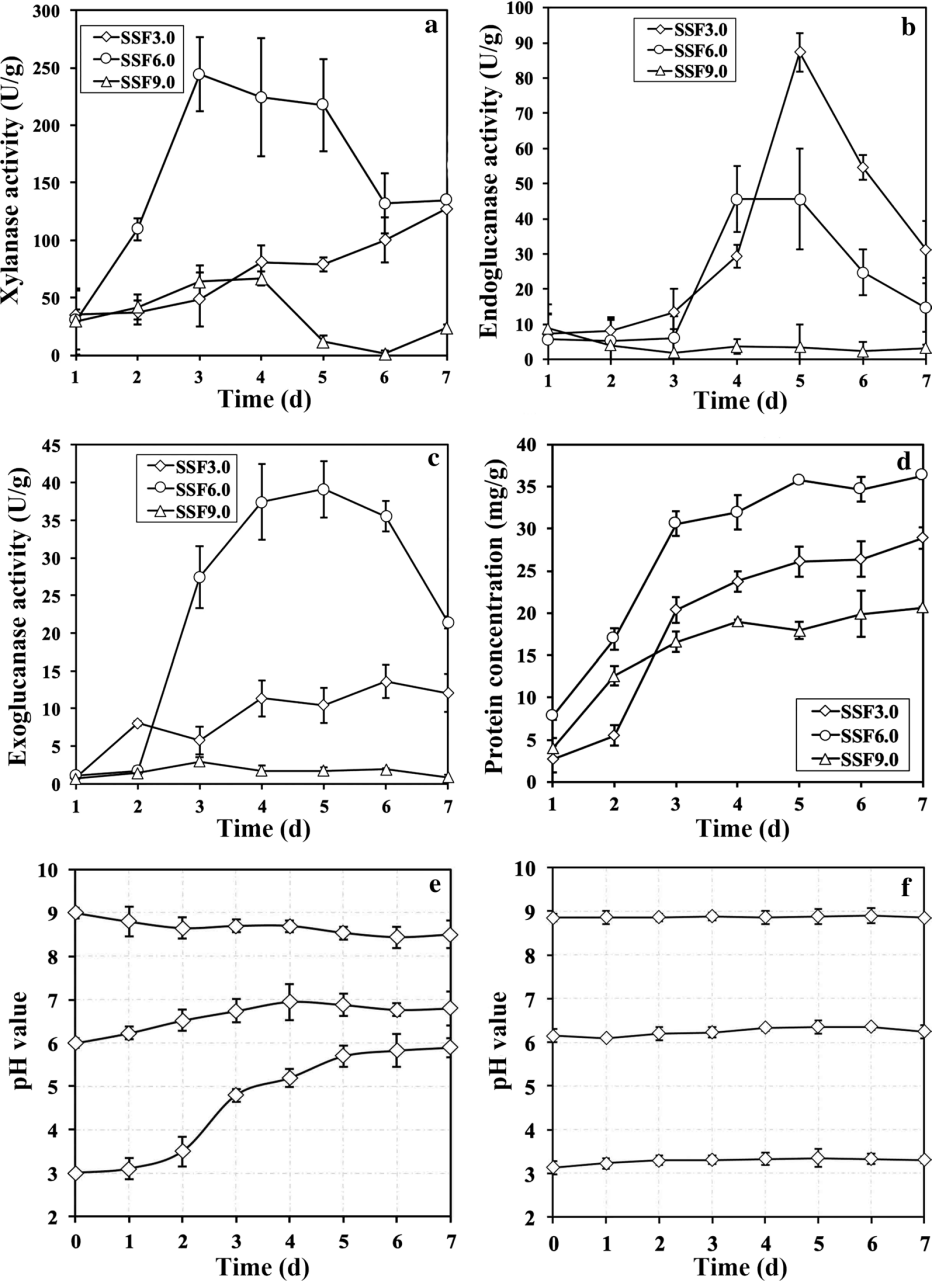
Enzyme activities and pH change in solid-state fermentations of rice straw by NJAU4742 strain under the different treatments of SSF3.0, SSF6.0 and SSF9.0. During the solid-state fermentation process, extracellular xylanase activities (**a**), extracellular endoglucanase activities (**b**), extracellular exoglucanase activities (**c**), extracellular protein concentrations (**d**) and environmental pH changes (**e**) were detected. The environmental pH changes when natural rice straw was degraded only by crude enzymes from NJAU4742 strain were also detected (**f**). The bars indicate the standard error of four replicates

The diverse growth conditions showed that different ambient pH values had strong effects on the NJAU4742 strain during the solid-state fermentation process of rice straw, and thus, it was very interesting to investigate the extracellular conditions of lignocellulose degradation in this process. Therefore, the treatments with initial pHs of 3.0, 6.0 and 9.0 (SSF3.0, SSF6.0 and SSF9.0) were chosen for more systematic analysis in detail. First, we detected the releasing amount of carbon dioxide (RCD) under these conditions, and the results (Fig. 2a) showed that the RCDs of SSF3.0, SSF6.0 and SSF9.0 all increased rapidly in the first 5 days. Then, the RCDs of SSF6.0 and SSF9.0 dropped rapidly to a similar final amount (3096 ppm and 2235 ppm, respectively) on the 7th day, but the decrease in RCD of SSF3.0 happened after the 6th day. For the values of RCDs, 3.91- or 1.41-fold changes were observed between the highest values of SSF6.0 and SSF9.0 or SSF3.0. These results revealed the following facts: (1) NJAU4742 strain grew the best and had the largest amount of biomass in SSF6.0 (Additional file 1: Figure S1); (2) sporulation was the predominant reason for the decrease in RCDs; (3) the growth cycle of SSF3.0 was clearly delayed when compared to that of SSF6.0 even though the biomass was equivalent. In addition, NJAU4742 strain in SSF9.0 also had a normal growth cycle but a limited increase in biomass, and this was assumed to be led by the low utilization efficiency of rice straw. The electron microscope analysis (Fig. 2b) indicated that rice straw in SSF9.0 still largely maintained a smooth surface similar to the rice straw without bio-fermentation (control check, CK), in comparison to the total destruction of the rice straw surface structure both in SSF3.0 and SSF6.0. Together with the fact that SSF9.0 also owned the lowest enzyme activities in all treatments (Fig. 3a–c, *p* < 0.01), including xylanase, endoglucanase and exoglucanase activities, and these results indicated that low utilization efficiency of rice straw in SSF9.0 seemed to be resulted by the low enzymatic hydrolysis of rice straw. To verify this viewpoint as mentioned above, we detected the concentrations of extracellular proteins (CEP) in all three treatments (Fig. 3d), and SSF9.0 indeed had the lowest values, but the differences (79% and 67% for SSF3.0 and SSF6.0, respectively) are not enough to completely support the hypothesis that a low amount of secreted enzymes led to an inefficient destruction of rice straw in SSF9.0. Therefore, it was hypothesized that the ambient pH of SSF9.0 was another main reason that largely inhibited all enzyme activities, as it is well known that most secreted cellulases and hemicellulases can only maintain approximately 20% of enzyme activity at pH 9.0 [20–22]. The crude enzymes of NJAU4742 strain induced by rice straw were detected to only have 19.13 ± 0.31% of xylanase activity and 1.12 ± 0.07% endoglucanase activity at pH 9.0 when compared to pH 5.0. Therefore, it is necessary to detect the ambient pH in SF9.0, and the results showed that there was no obvious change in pH during the 7-day fermentation (Fig. 3e). This high pH of 9.0 extremely affected extracellular enzyme activities and stabilities, and thereby strongly contributed to the growth limitation of high pH. Interestingly, 3.0 was also a value far from the optimal pH of most lignocellulases, but the rice straw in SSF3.0 was effectively utilized. The change profile of pH value in SSF3.0 illustrated that the pH value was rapidly and largely increased as the fermentation continued until reaching a final value of 5.8 (Fig. 3e), which was very close to the optimal pH for most lignocellulases secreted by acidic filamentous fungi. Finally, the pH increases in SSF6.0 was also observed, which indicated that the growth of NJAU4742 strain could significantly eliminate the high concentration of H^+^ in two ways: (1) neutralized by the intermediate products released from the rice straw; (2) absorbed and utilized by NJAU4742 strain, or offset by the secretions from NJAU4742 strain. In fact, the results in Fig. 3f excluded the first mechanism, and thus indicated that the second mechanism is responsible for the pH increase during the solid-state fermentation process.

### Proteomic analysis for solid-state fermentations of NJAU4742 strain at pH 3.0, 6.0 and 9.0

The different growth conditions and enzymatic properties above showed that diverse regulations must exist in the solid-state fermentations of NJAU4742 strain at different initial pH values, thus proteomic analysis was used to investigate the details of responding to ambient pH by NJAU4742 strain. After protein extraction, the mycelial proteins and extracellular proteins were mixed based on the protein concentration (mg g^−1^ dw, protein/substrates), meaning that both were extracted from the same amount of the fermented materials (see “Methods”). SDS-PAGE analysis showed that the mixed total proteins were repeatable in each treatment and that significant differences existed among SSF3.0, SSF6.0 and SSF9.0 (Additional file 1: Figure S2). Consistent with the highest enzyme activities, total proteins of SSF6.0 had the most abundant protein bonds, as shown in Additional file 1: Figure S2. After quality control, total proteins from SSF3.0, SSF6.0 and SSF 9.0 were separately detected and analyzed using the SWATH technique, which resulted in a total number of 1139 proteins when using the whole protein sequences of NJAU4742 strain as the database.

### Statistical analysis

For all the determined proteins, their coverage rate of peptides was calculated and seemed to fit a noncentral F-distribution (Additional file 1: Figure S3A), with approximately 90% of identified proteins having a coverage rate of peptides lower than 37%. The protein abundances in all samples (SSF3.0, SSF6.0 and SSF9.0) conformed to a normal distribution with a range of 10^3^–10^8^ (Additional file 1: Figure S3B). Together with the considerable repeatability of each sample (Additional file 1: Figure S3C) and objective statistical information produced with SWATH analysis (data not shown), these data of the identified proteins allowed the deep analysis of the responses to different ambient pH values by NJAU4742 strain.

### Protein location analysis

**Fig. 4:**
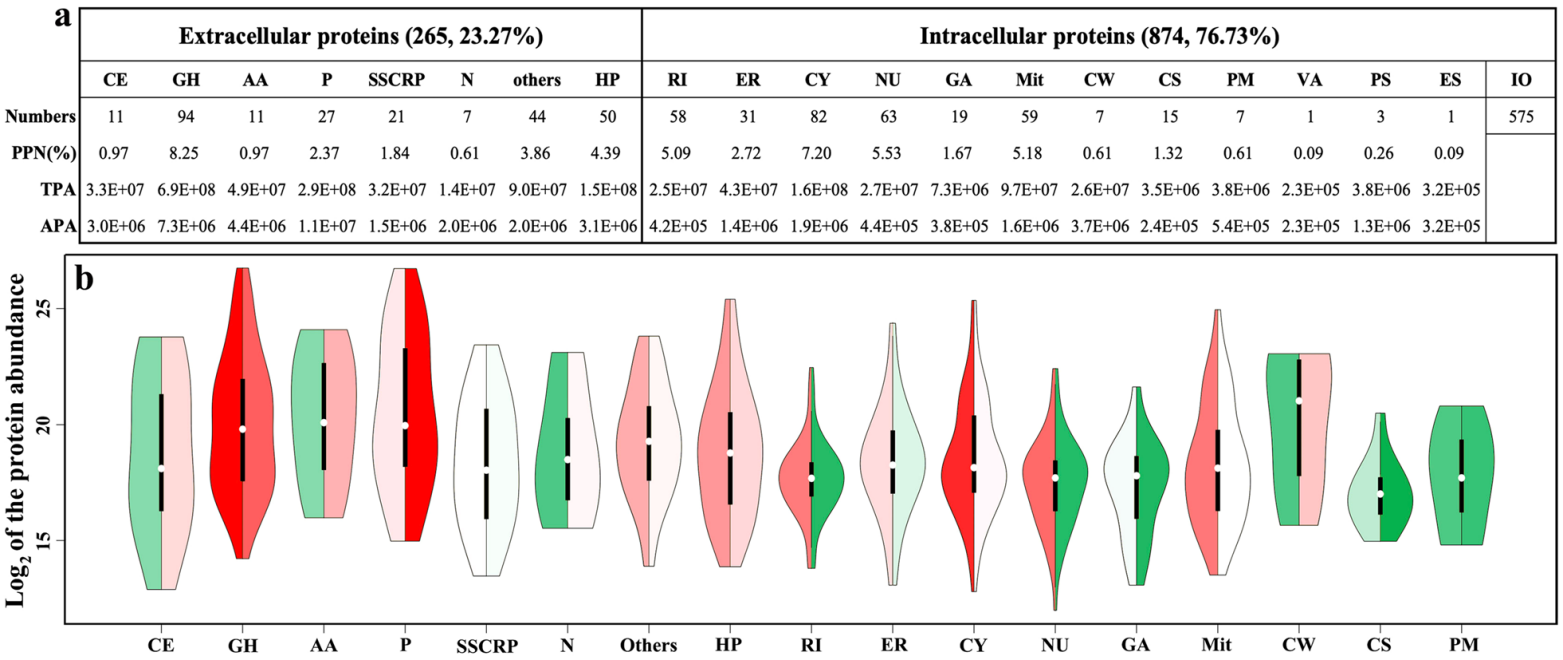
The location and distribution of the identified proteins by SWATH analysis. **a** The statistical information for each grouped protein. **b** Violin plot of protein abundances for each grouped protein. Colors (red, higher values; green, lower values) on the left half and the right half stand for the total numbers and the average protein abundance, respectively. *CE* carbohydrate esterase, *GH* glycoside hydrolase, *AA* auxiliary activity, *P* protease, *SSCRP* small secreted cysteine-rich protein, *N* nuclease, *others* other functional extracellular proteins, *HP* hypothetic protein, *RI* ribosome, *ER* endoplasmic reticulum, *CY* cytoplasm/cytosol, *NU* nucleus, *GA* golgi apparatus, *Mit* mitochondria, *CW* cell wall, *CS* cytoskeleton, *PM* plasma membrane, *VA* vacuole, *PS* peroxisome, *ES* endosome, *IO* intracellular others, *PPN* the percentage of protein numbers, *TPA* the total protein abundance, *APA* the average protein abundance

All 1139 identified proteins were classified based on their locations (Fig. 4 and Additional file 2), in which 921 proteins were annotated as intracellular proteins and 346 proteins therein were located in different detailed intracellular regions, such as the ribosome (58 proteins), endoplasmic reticulum (31 proteins), cytoplasm/cytosol (82 proteins), golgi apparatus (19 proteins), mitochondria (59 proteins), and nucleus (63 proteins). Additionally, 265 proteins were annotated as extracellular proteins, including 94 glycoside hydrolases, 11 carbohydrate esterases, 11 auxiliary activities enzymes, 27 proteases, 21 small secreted cysteine-rich proteins (SSCRPs), 7 nucleases, 44 functional proteins and 50 unknown proteins. These identified proteins had a very uneven distribution of expression abundances. For example, though less than a quarter of the total number of proteins, the 265 extracellular proteins amazingly shared > 58.2% of total protein abundances, and did not conform to the normal distribution. In contrast, most intracellular organelles fitted a much greater distribution of spindle morphology in protein abundance when compared to extracellular proteins (Fig. 4). These findings indicated that the secreted proteins from NJAU4742 strain did not follow intracellular rules, but continually secreted enzymes to achieve much more nutrition by degrading rice straw in a resource-poor environment. From this aspect, glycoside hydrolases and proteases evidently played important roles in their > 72% of extracellular protein abundances, which showed the critical need for carbon and nitrogen sources by strain NJAU4742 strain.

### CAZymes analysis

**Fig. 5:**
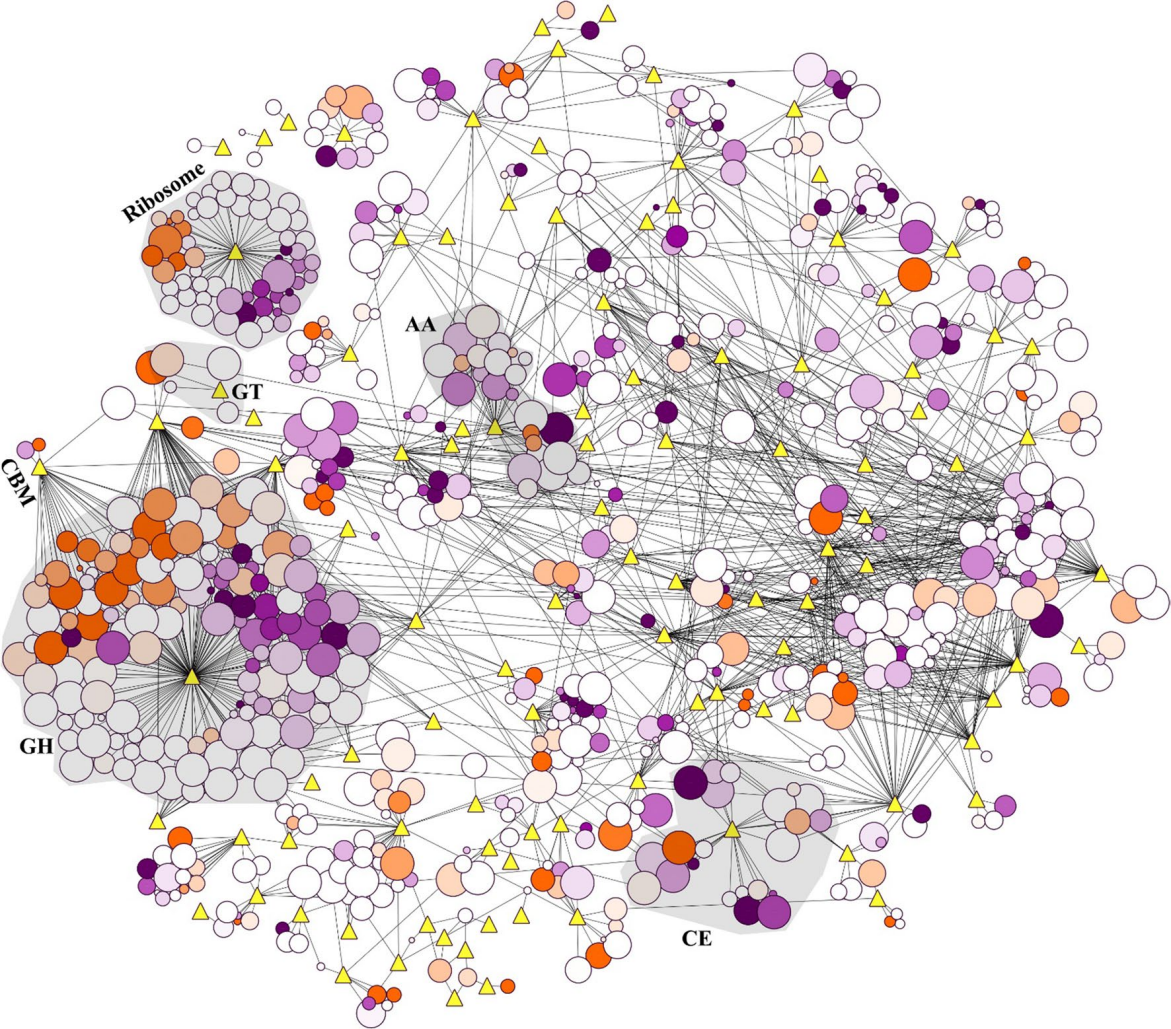
Protein expression differences between SSF3.0 and SSF6.0 visualized as a Cytoscape interaction network. Nodes are proteins (circles) or KEGG/carbohydrate-active enzyme (CAZy) categories (yellow diamonds); edges are protein interactions defined by KEGG or CAZy databases. Protein node sizes indicate the log_10_ of protein expression values. Node colors represent the fold change (orange, up-regulated; purple, down-regulated) of protein expression values between SSF3.0 and SSF6.0

Rice straw, a natural lignocellulose, mainly consisted of cellulose, hemicellulose and lignin. In solid-state fermentation, rice straw was efficiently deconstructed by the secreted enzymes, which were usually classified into different families in the CAZyme database, including glycoside hydrolases (GHs), polysaccharide lyases (PLs), carbohydrate esterases (CEs), auxiliary activities (AAs) and carbohydrate-binding modules (CBMs). Unfortunately, no PLs were identified in this experiment, even though they have positive functions in degrading rice straw. In Fig. 5, Additional file 1: Figure S4 and Additional file 3, 190 identified CAZymes (GHs, CEs, AAs and CBMs), and 510 other KEGG pathway-annotated proteins were used to construct a protein network, which significantly showed the functions of pH in regulating these identified proteins. For enzymes in the CEs and AAs, the treatment of SSF6.0 had a relatively higher inductive effects when compared to those of the treatments of SSF3.0 and SSF9.0. For enzymes in the GHs, however, the result became complex. When comparing SSF3.0 and SSF6.0, differential proteins were divided into two numerically comparable groups (the up-regulated and the down-regulated, 1.5-fold change and *p* < 0.05), which meant that SSF3.0 or SSF6.0 could selectively enhance or inhibit the inductions of some enzymes in GHs. This phenomenon did not happen in the comparison between SSF9.0 and SSF6.0, where nearly all differential proteins had high abundances in SSF6.0.

**Fig. 6:**
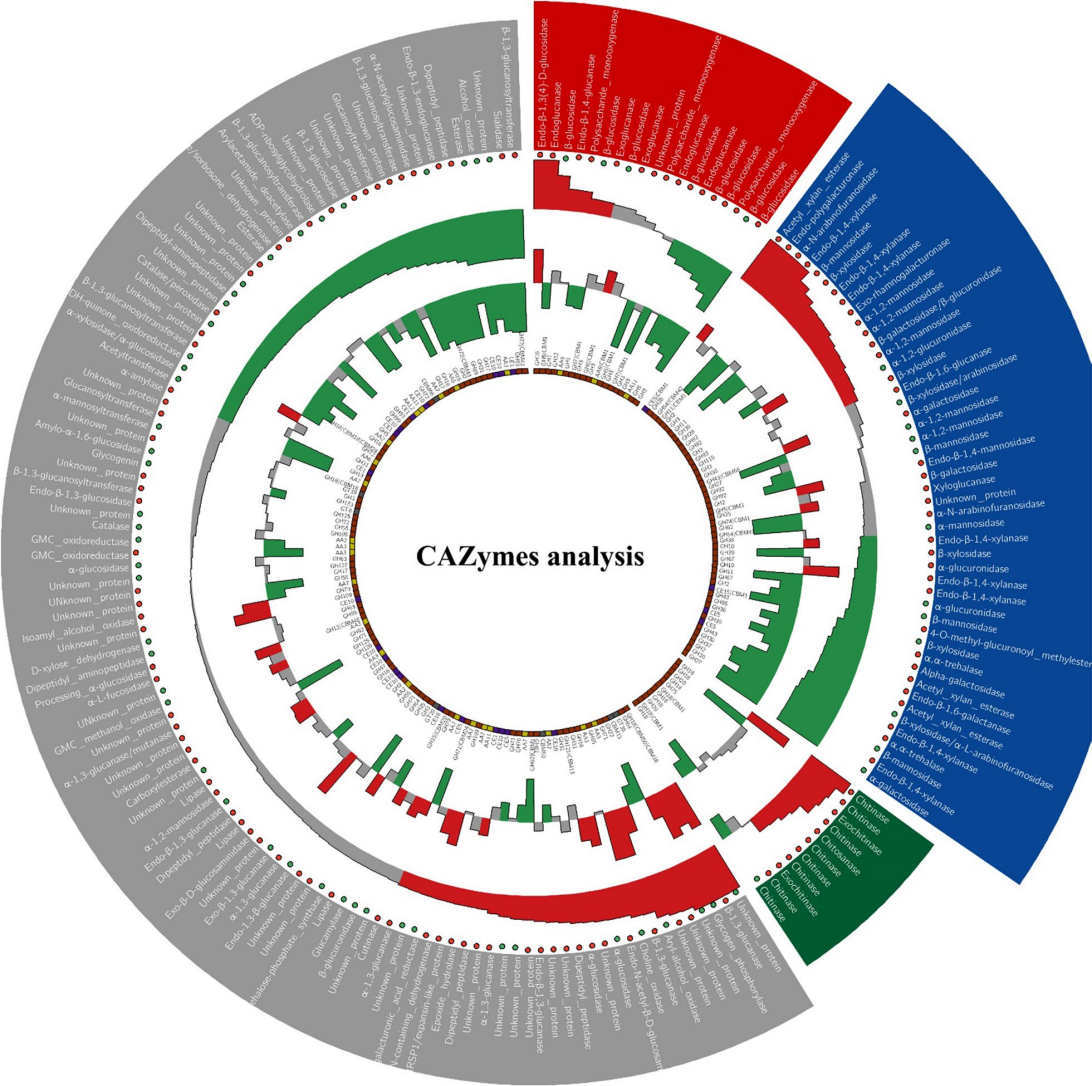
CAZymes analysis among SSF3.0, SSF6.0 and SSF9.0. The first (outermost) circle represents different grouped proteins and their descriptions (red, cellulases; blue, hemicellulases; gray, other proteins). The second circle shows the condition of the signal peptide (red circle, yes; green circle, no). The third and fourth circles show the fold changes of protein abundances of SSF3.0/SSF6.0 and SSF9.0/SSF6.0, respectively. Red, fold change ≥ 1.5; green, fold change ≤ 2/3; gray, 2/3 < fold change < 1.5. The innermost circle gives the family annotations for each protein

The identified CAZymes included cellulases, hemicellulases, chitinases and other functional enzymes (Fig. 6). Among cellulases, 5 endoglucanases (families: GH5, GH7, GH12 and GH16), 2 exoglucanases (GH6 and GH7), 8 β-glucosidases (GH1, GH3 and GH5) and 3 polysaccharide monooxygenases (AA9 and AA11) were identified. For hemicellulases, 8 endoxylanases (GH10, GH11 and GH30), 6 β-xylosidases (GH3, GH39 and GH43), 8 hydrolytic enzymes of xylan side chains (GH54, GH67, CE5 and CE15), 10 mannanases (GH2, GH5, GH38 and GH92) and 14 other functional hemicellulases (GH2, GH27, GH28, GH30, GH35, GH36, GH37, GH62, GH65, GH74 and GH115) were identified. Additionally, 11 chitinases (GH18, GH20 and GH75) and 113 other enzymes (including glucanases, glucosidases, esterases, and unknown proteins) were also identified. As shown in Fig. 6, the up different inductions by pH values were also reflected in these subgroups (cellulases and hemicellulases) of GHs when comparing SSF3.0 and SSF6.0. That is, some cellulases/hemicellulases were highly expressed in SSF3.0, and some other cellulases/hemicellulases were highly expressed in SSF6.0. For those cellulases/hemicellulases down-regulated in SSF3.0, they were also mostly down-regulated in SSF9.0 when compared to SSF6.0 (1.5-fold change and *p* < 0.05). However, it is different for chitinases, where SSF3.0 seemed to be the best induction condition in all treatments. For these rice straw-degrading-related CAZymes, different induction performances evidently existed, including (1) highest expression abundances in SSF3.0; (2) highest expression abundances in SSF6.0; (3) lowest expression abundances in SSF6.0 and (4) no obvious differences at all pH values. All these conditions clearly indicated that different ambient pH values functioned to regulate the secretion of various lignocellulases.

**Fig. 7:**
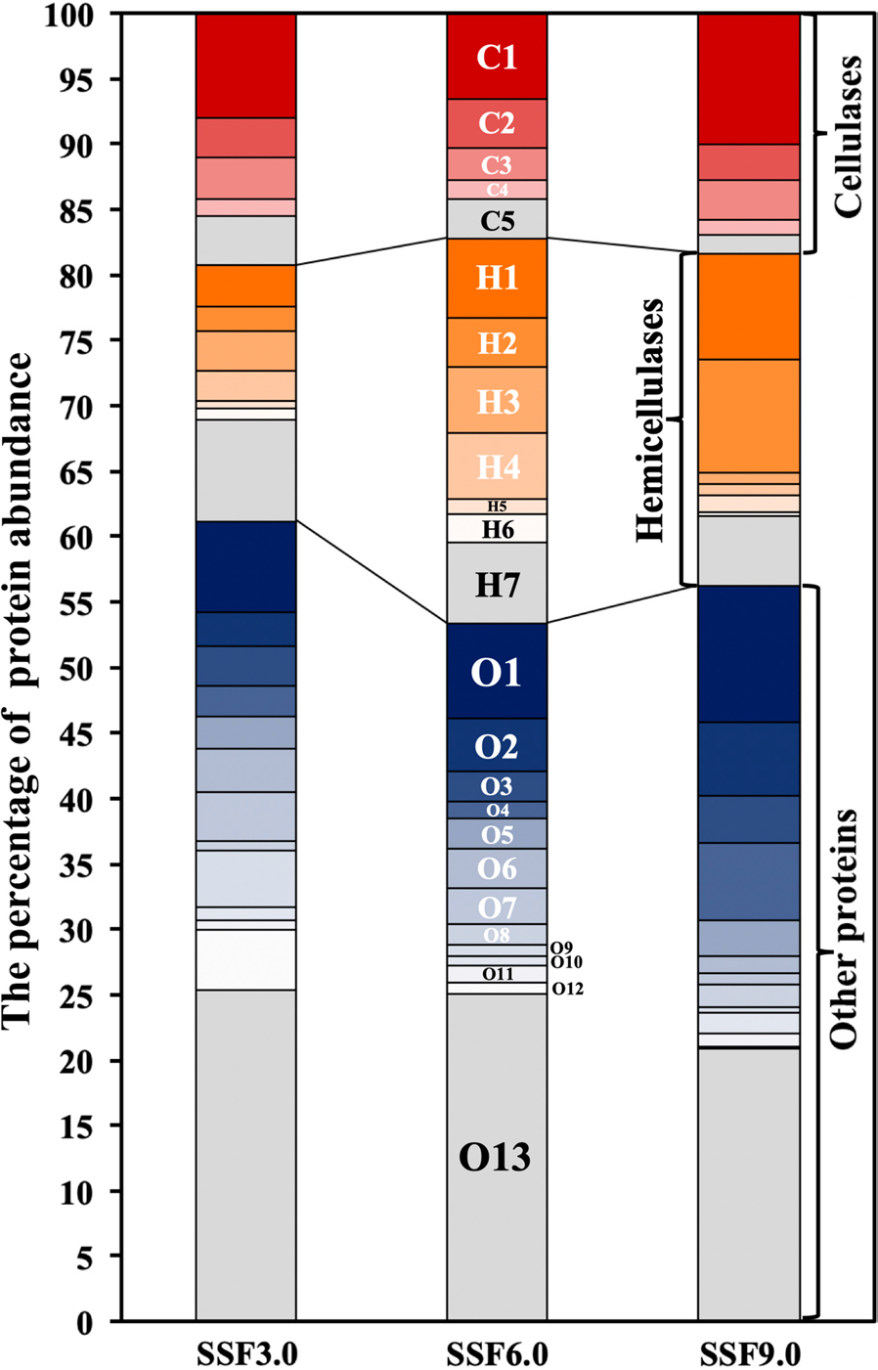
The distribution of extracellular protein abundances. The extracellular proteins with percentage ≥ 1% of total secreted proteins are shown in the figure. C1, exoglucanase (OPB43438); C2, β-glucosidase (OPB36250); C3, exoglucanase (OPB45635); C4, polysaccharide monooxygenase (OPB41190); C5, other cellulases; H1, endo-β-1,4-xylanase (OPB45659); H2, endo-β-1,4-xylanase (OPB40690); H3, α-*N*-arabinofuranosidase (OPB41212); H4, endo-β-1,4-xylanase (OPB43840); H5, β-galactosidase (OPB41632); H6, α-1,2-mannosidase (OPB36783); H7, other hemicellulases; O1, neutral protease (OPB36603); O2, xyloglucanase (OPB46497); O3, hypothetical protein (OPB44171); O4, hypothetical protein (OPB41390); O5, aspartyl protease (OPB39078); O6, elastinolytic metalloproteinase (OPB40519); O7, α-1,3-glucanase (OPB40799); O8, leucine aminopeptidase (OPB40843); O9, subtilisin-like protease (OPB41391); O10, glutaminase A (OPB43941); O11, hypothetical protein (OPB41382); O12, trypsin-like protease (OPB36132); O13, other proteins

When focusing on the protein abundances of those secreted CAZymes, very significant differences existed (Fig. 7). Among cellulases, the most abundant enzymes, including 2 exoglucanases, 1 β-glucosidase and 1 polysaccharide monooxygenase, composed 15.6% of all extracellular proteins in protein abundance. Among hemicellulases, four abundant enzymes (3 endoxylanases and 1 α-*N*-arabinofuranosidase) composed 16.2% of all extracellular proteins in protein abundance. Meanwhile, a xyloglucanase and a α-1,3-glucanase had percentages of 4.1% and 2.4%, respectively. These results reflected that some enzymes in CAZymes were the universal choices among filamentous fungi, while their largely unequal secretions possibly satisfied the need for rapid degradation of rice straw. Certainly, protein abundances could not decide the enzyme functions, but their differential distribution here actually showed that a natural proportion of these secreted functional enzymes was reasonable for degradation of the lignocellulose.

## Discussion

In solid-state fermentation, NJAU4742 strain had adapted to a wide environmental pH condition, ranging from 3.0 to 9.0, and showed a distinct preference for the specific pH of 6.0. Beyond adaptation, NJAU4742 strain could also significantly reconstruct the environmental pH condition by alkalinization, in which the pH value was elevated from 3.0 to 6.0 in 5 days. Therefore, it is very interesting to discuss NJAU4742’s alkalinization, which is hence hypothesized to be the result of three possible mechanisms: (I) the released components from the degradation of rice straw neutralized the acidic environmental pH; (II) NJAU4742 strain absorbed extracellular H^+^ and (III) the secretions of NJAU4742 strain elevated the environmental pH. For hypothesis I, the main components of rice straw contained polysaccharides (cellulose, xylan and mannan) and lignin, however, the released basic units, such as oligosaccharides, glucose, xylose, mannose and aromatic alcohols, actually had little influence on the ambient pH. The components that could alter the ambient pH were pectin and those substituent groups of hemicellulose, of which the complete hydrolysis would release various organic acids, including galacturonic acid, glucuronic acid, ferulic acid, *p*-coumalic acid, and acetic acid [7]. Meanwhile, protein degradation by extracellular proteases could also release various amino acids from the substrate. Apparently, these produced organic acids will function as a reverse to the environmental alkalinization by NJAU4742 strain, and were probably absorbed and utilized rapidly by NJAU4742 strain, much like the produced sugars, and this behavior functioned in regulating ambient pH, which belonged to part of hypothesis II (absorbing extracellular acids). Additionally, the original high concentration of H^+^ in SSF3.0 strongly drew attention to an enzyme called the H^+^-ATPase, which was identified and significantly regulated by pH in this study (OPB40853, *p* < 0.05, Additional file 2). This enzyme was generally located on the plasma membrane of plants and fungi, and belonged to the P-type cation-translocating ATPase family. Many previous studies showed that H^+^-ATPases were always regulated by glucose, an acidic pH and weak organic acids [23], which existed in solid-state fermentations of NJAU4742 strain and resulted in the highest expression level of the H^+^-ATPase in SSF6.0. The complex regulation of the H^+^-ATPase in the SSF of NJAU4742 strain suggested its relationship with NJAU4742 strain alkalinization, but may also work as a counter for its reported function of pumping H^+^ out of the cell to create a transmembrane electrochemical proton gradient for nutrient uptake and stabilizing intracellular pH [24, 25]. Actually, under such a low pH of 3.0 condition (high natural potential across plasma membrane), it was hard to know whether the H^+^-ATPase was still needed to work or if there were any possible patterns of the H^+^-ATPase helping NJAU4742 strain absorb H^+^ in some way, which still required a more in-depth study. For hypothesis III, many studies have proposed that fungi could modify the pH of their environment by secreting acids or alkalis. For example, *Sclerotinia sclerotiorum* and *Botrytis* sp. could produce oxalic acid [26], while *Penicillium* sp. and *Aspergillus* sp. can secrete mainly gluconic and citric acids [27, 28]. Although pH was slightly dropped in SSF9.0, it was still difficult to judge whether NJAU4742 strain secreted acids in this study, but at least this was not the main determinant for the acidic environment. Some other fungi, such as *Colletotrichum gloeosporioides* [29], *Dictyostelium discoideum* [30] and *Debaryomyces hansenii* [31], and especially for *Candida albicans* [32], could alkalinize their environment by secreting ammonia, a byproduct during the amino acid metabolic process. By a BLAST search, the homologs of an ammonium transporter of MEPB and three ammonia transporters (ATOs) in this process were found in strain NJAU4742, but no homologs were detected for the critical regulator (Stp2p) and amino acid catabolic enzyme (Ach1p). Among the four detected homologs, only one ATO protein (OPB44235) was identified by SWATH, but its highest expression level in SSF6.0 could not be explained logically. These previous reports of other fungi’s alkalinization supported that NJAU4742 strain in this study might elevate the ambient pH by secreting another kind of alkali (not ammonia), which was entirely possible because *C. albicans* also shared multiple mechanisms of environmental alkalinization not only ammonia secretion [32–34]. Certainly, H_2_O_2_ and secondary metabolites were also considered as the possible functional parts of the complex environmental alkalinization of NJAU4742 strain.

In SSF3.0, the growth inhibition of NJAU4742 strain by the low pH was relieved as the ambient pH increased, which indicated that environmental alkalinization determined the growth ability of NJAU4742 strain at pH 3.0. This fact reflects that a complex regulatory system exists for detecting different pH values and further, responding to the pH. The results here clearly showed that extracellular proteins, such as cellulases, hemicellulases and chitinases, were under the regulation of this possible system. Similarly, some cellulase and xylanase genes were also reported to be controlled by the ambient pH in *T. reesei* [35], *Humicola grisea* [36] and *Neurospora crassa* [19], and thus together showed the universality of pH regulation of lignocellulases in filamentous fungi. Actually, there are already some reports that the integrity of seven proteins, designated PacC, PalA, PalB, PalC, PalF, PalH and PalI, were responsible for gene regulation at different pH values [14]. Under acidic growth conditions, PacC^72^ (72 kDa) is inactive without any known role in transcriptional regulation, but it is converted to PacC^53^ (53 kDa) and further to PacC^27^ (27 kDa), which is subsequently active and functions in the activation of alkaline-expressed genes and repression of acid-expressed genes. PacC orthologs were found to modulate the expression of cellulolytic genes in response to ambient pH in filamentous fungi, but the effects varied depending on the organisms. For example, deletion of the *pacC* gene in *T. reesei* significantly increased the cellulolytic enzyme activities at neutral pH [18], but the transcriptome analysis showed only a few cellulase- and hemicellulase-encoding genes were clearly under PacC regulation [16]. In *Aspergillus nidulans* [37], PacC significantly upregulated the expression of cellulolytic genes at alkaline pH, but had a reverse observation in *N. crassa* [19]. The homologs of the seven proteins in the whole PacC-mediated system could be detected and encoded in the genome of NJAU4742 strain, and thus suggested that those pH-regulated lignocellulases were possibly under the control of this system. Although none of the homologs appeared in the 1139 identified proteins, further study of PacC-mediated and other possible systems in regulating extracellular enzymes by the ambient pH, will be very interesting.

Obviously, filamentous fungi regulated the secretion of extracellular enzymes by the ambient pH, however, we need to understand the biological significance behind this behavior. The organisms are highly evolved, and thus the secretion adjustment of extracellular enzymes at different pH values is believed to be an adaptive mechanism to ensure better survival in that environment. That means, NJAU4742 strain possibly achieved more efficient degradation of rice straw by adjusting the differential secretion of cellulases and hemicellulases when exposed under the different pH values. Logically, the adjustment is to achieve the optimal synergy of extracellular enzymes under different pH conditions, which possibly could be explained from the different enzymatic properties of cellulases and hemicellulases. And, it is really the fact that cellulases and xylanases from the same filamentous fungus have different enzymatic properties, such as thermostability, optimal temperature, specific activity and functional domains [38]. Just like extracellular proteases, they were distinguished as acidic, neutral and alkaline proteases and regulated by the PacC-mediated system [39]. The secreted proteases of NJAU4742 strain also showed significant pH regulation in this study (Additional file 2). Besides, Xiong et al. [40] reported that xylanase I was most secreted and active at low pH value (4.0), while xylanase III was most secreted and active at high pH value (6.0) in *T. reesei*. Xylanase II was clearly produced at both pH values. These results and analysis clearly support such a judgment that the pH-dependent regulation of extracellular enzymes is probably for achieving higher enzymatic synergy by secreting the optimal enzymes at different pH values, so as to obtain a most efficient degradation of lignocellulose. Certainly, more detailed and in-depth researches need to be carried out to explain this pH-dependent synergy of lignocellulases.

Additionally, we also noticed the stable differential distribution of extracellular enzyme abundances, which was believed to be the result of evolution, as well as a kind of synergistic performance of multiple enzymes. For cellulose degradation, exoglucanase attacked the natural and LPMO-produced ends of crystalline cellulose, and then the products could be hydrolyzed into glucose by β-glucosidase. The high abundance of these three functional enzymes suggested the specific enhancement of the main steps in the degradation of crystalline cellulose, but not including the endoglucanases, which seemed to have difficulties when attacking the middle of the cellulose chain in the crystalline structure. In contrast, endoxylanase was the largest abundant secreted enzyme among all xylanases, corresponding to the fact that endoxylanase could easily attack the whole chain of xylan for its non-crystalline structure. Facing the complex 3D structure of lignocellulose, the differential distribution of extracellular enzymes is indeed likely to be for greater synergy efficiency, and further analyzing this synergy will be very interesting and valuable in biofuel industry.

## Conclusions

This study described the performances of NJAU4742 strain during the submerged and solid-state fermentation processes at different initial pH values in detail. The results showed that ambient pH dominated the growth, sporulation, extracellular enzyme activities and decomposition rate of rice straw by NJAU4742 strain. Proteomic analysis deeply revealed the intracellular and extracellular proteins during the solid-state fermentation processes and clearly quantified 190 functional enzymes for rice straw degradation especially for their extracellular synergetic distribution. More importantly, the pH-regulated lignocellulose-degrading enzymes in filamentous fungi were firstly distinguished and classified in detail. All these results contribute to the theoretical basis for the degradation of plant biomass by filamentous fungi in the biofuel and biological organic fertilizer industries.

## Methods

### Strains and culture conditions

*Trichoderma guizhouense* NJAU4742 was isolated from a mature compost sample and stored in 15% glycerol at − 80 °C in our lab, and its genome sequence was already published in the NCBI database (Accession No. LVVK00000000.1). Murashige and Skoog (MS) medium (1.36 g/L KH_2_PO_4_, 2.13 g/L Na_2_HPO_4_, 0.2 g/L MgSO_4_·7H_2_O, and trace elements) was used as the liquid medium for cultivating NJAU4742 in both submerged and solid-state fermentation, but varied in percentages of rice straw, which were 2% (w/v) or 25% (w/v), respectively. The rice straw used in this study was obtained from the local farmland (Nanjing, China) and then washed with running water for 48 h, after which it was dried entirely at 40 °C for 5 h and ball-milled into powder using a ball-miller (Retsch MM400, Germany) with ZrO_2_ balls (15 mm, diameter) and vessel (35 mL, volume), and the parameters were set as follows: 15 Hz, 1 min milling followed by a 30-s pause, with a total time of 5 min, after which the rice straw powder was screened by a 30 mesh sieve with the average size less than 0.6 mm. The initial pH values of the submerged or solid-state fermentations were adjusted by adding 0.5 M H_2_SO_4_ or NaOH and monitored with a pH meter (PB-10, Sartorius, Germany).

Spores of NJAU4742 strain were collected from the incubated PDA plates by washing followed by the filtration through 4 layers of gauze. After two repeated washing by the sterilized ddH_2_O, the spore concentration was subsequently adjusted to be 1 × 10^7^/mL through the method of hemocytometer counting. For the submerged fermentation, a total of 5 × 10^6^ spores were inoculated into 50 mL MS medium with 2% (w/v) rice straw in a 250-mL triangular flask and grown under the condition of 28 °C and 150 rpm. For the solid-state fermentation, a total of 1 × 10^7^ spores were inoculated into a 500-mL triangular flask containing 75 mL MS medium and 25 g rice straw, and grown under the condition of 28 °C. All treatments were performed for total 7 days, and sampled at the incubation times of 1, 2, 3, 4, 5, 6 and 7 days. Three biological repeats existed for each sampling point.

### Enzyme activity assays

Endoglucanase, exoglucanase and xylanase activities were measured using carboxymethyl cellulose sodium (CMC-Na) (Sinopharm Chemical Reagent Co., China), *p*-nitrophenyl-β-d-cellobiose (*p*NPC) (Sigma, USA), and oat spelts xylan (Sigma, USA) as the substrate, respectively, according to the methods described previously [20, 41]. One unit of enzyme activity was defined as the amount of enzyme required to release 1 μmol reducing sugars or *p*NP from the substrate in 1 min. Protein concentrations were determined using the Bradford protein assay kit (ThermoFisher, USA) based on the product description [42]. All the enzyme activities were reported as the means of at least three replicates.

### Electron microscope analysis and respiratory rate determination

After fixation with 2.5% glutaraldehyde for 2 days, the samples from different treatments were immediately dehydrated in 10 mL of ice-cold 200 proof ethanol (Sigma-Aldrich), point-dried in a critical point dryer (HCP-2, Hitachi High-Technologies Corporation, Japan) and coated with 60% Au/Pd in a sputter coater (Sputter Coater Baltec SCD500, Bal-Tel) [43]. The surface morphologies of different samples were observed by using an S-4800 II field emission scanning electron microscope (Hitachi, Japan). The respiratory rate of NJAU4742 strain during the solid-state fermentation process was evaluated by tracking the release of CO_2_. Briefly, the triangular flasks of solid-state fermentations were sealed with a parafilm 2 h before sampling. When sampling, 20 mL of gas in the triangular flask was extracted with a syringe and then injected into the gas sampling bag (E-SWITCH, China), and the contents of CO_2_ was determined by a gas chromatography (Agilent 7890A) equipped with Porapak Q column and a flame ionization detector (FID) according to Zhang et al. [44] with some modification: 500 µL of gas were injected through a septum with the temperature of 80 °C, and the carrier gas (N_2_) flow-rate was 30 mL min^−1^; meanwhile, the temperature of the methanizer and detector were set as 450 °C and 250 °C, respectively.

### Protein extraction and SWATH analysis

Samples of each treatment in solid-state fermentations at the 4th day were taken and used to extract proteins for proteome analysis. Extracellular protein extraction was carried out as follows: 150 mL deionized ddH_2_O was added into triangular flasks, which were then shaken at 180 rpm for 1 h, and the supernatants were collected by centrifugation at 10,000 rpm for 10 min and filtration through a 0.22 µm sterile membrane. The precipitates of the former step were used to prepare the mycelial intracellular proteins, which were extracted by using the NoviPure^®^ Soil Protein Extraction Kit (MOBIO, USA) according to the manufacturer’s protocol. After that, extracellular and intracellular proteins, both extracted from the same amount of fermentation materials samples (mg g^−1^ dw, protein/substrates), were directly mixed and concentrated by lyophilization and then stored at − 80 °C. Two biological repeats existed for each condition in proteomic analysis.

The whole SWATH analysis was commissioned to a specialized company (GeneCreate, Wuhan, China), and the analysis is described briefly as follows: 100 μg of proteins in each sample were digested by trypsin (Promega, USA) at 37 °C overnight, separated by the Strata-X C18 pillar and dried using a vacuum concentration meter. LC–ESI-MS/MS analysis was performed on an AB SCIEX nanoLC–MS/MS (Triple TOF 5600 plus) system in two phases: data-dependent acquisition (DDA) was followed by SWATH acquisition, where all samples were mixed and detected in DDA, and the resulting data were used as a library for analysis of each sample by SWATH. Spectral library generation and SWATH data processing were performed using skyline version 3.5 software [45] complying with the following rules: (i) peptides containing modifications and/or shared by different protein entries/isoforms were excluded from selection; (ii) peptides having a ProteinPilot-identified confidence less than 95% were excluded; (iii) up to five fragment ions that were ranked by intensity were chosen; (iv) fragment ions within the SWATH isolation window were excluded from selection; and (v) to control false discovery rate, a random mass shift of Q1 and Q3 m/z strategy [46] was used to create a decoy spectra library for targeted peak extraction, a mass-to-charge tolerance of at most 10 ppm (0.05 m/z) was allowed for both peptide precursor and fragment ion. Fragment ion areas that belonged to one peptide were added to obtain a peptide’s abundance, and the total abundance of peptides for a given protein was determined to obtain the protein’s abundance. To eliminate the random errors and sample bias, all the data among samples were normalized using the median normalization method [47], and the mProphet algorithm was used both for sample normalization and for assessing the data confidence [48].

### Bioinformatic analysis

CAZymes annotation was carried out using the hmmscan tool from HMMER software [49] to search the dbCAN database [50], and the output was processed by the script of hmmscan-parser.sh (https://github.com/carden24/Bioinformatics_scripts/blob/master/hmmscan-parser.sh). KEGG pathway annotation was performed using KEGG Orthology-Based Annotation System 2.0 (KOBAS) [51]. Gene location was manually annotated by combining information from GO Ontology, KEGG annotation, NCBI gene descriptions and some previous articles. Signal peptide was predicted using SignalP 4.0 software [52]. Circos [53] and Cytoscape [54] were used for data visualization. Violin plot was performed using the R package vioplot [55].

## Supplementary information

**Additional file 1: Table S1.** SWATH results of the identified proteins in SSF3.0, SSF6.0 and SSF9.0. **Figure S1.** Growth conditions of NJAU4742 strain in SSF2.0, SSF3.0, SSF6.0, SSF8.0 and SSF9.0. **Figure S2.** 2D SDS-PAGE of the total proteins in SSF3.0, SSF6.0 and SSF9.0 for SWATH analysis. **Figure S3.** Statistical analysis of SWATH results. After SWATH detection, Frequency distribution of protein coverages and protein abundances were shown in (**A**) and (**B**), respectively. Protein abundance from each repeat was plotted and compared in (**C**). **Figure S4.** Protein expression differences between SSF9.0 and SSF6.0 visualized as a Cytoscape interaction network.

**Additional file 2.** The identification and quantification results of all the proteins from different treatments.

**Additional file 3.** The raw data for the Cytoscape interaction network analysis.

## Data Availability

Not applicable.

## Abbreviations

GH: glycoside hydrolase
PL: polysaccharide lyase
CE: carbohydrate esterases
AA: auxiliary activities
CBM: carbohydrate-binding module
RCD: the releasing amount of carbon dioxide
SSF: solid-state fermentation
CEP: the concentrations of extracellular proteins
