# Supplementary material for: Proteomic analysis reflects an environmental alkalinization-coupled pH-dependent mechanism of regulating lignocellulases in Trichoderma guizhouense NJAU4742
Source: Biotechnol Biofuels. 2020 Jan 11;13:6. doi: 10.1186/s13068-020-1651-0 (PMC6954547; doi:10.1186/s13068-020-1651-0)
Supplement: Supplementary file 1 — Additional file 1: Table S1. SWATH results of the identified proteins in SSF3.0, SSF6.0 and SSF9.0. Figure S1. Growth conditions of NJAU4742 strain in SSF2.0, SSF3.0, SSF6.0, SSF8.0 and SSF9.0. Figure S2. 2D SDS-PAGE of the total proteins in SSF3.0, SSF6.0 and SSF9.0 for SWATH analysis. Figure S3. Statistical analysis of SWATH results. After SWATH detection, Frequency distribution of protein coverages and protein abundances were shown in (A) and (B), respectively. Protein abundance from each repeat was plotted and compared in (C). Figure S4. Protein expression differences between SSF9.0 and SSF6.0 visualized as a Cytoscape interaction network. [file 13068_2020_1651_MOESM1_ESM.docx]

**Additional data for**

**Proteomic analysis reflects an environmental alkalinization coupled pH-dependent mechanism of regulating lignocellulases in *Trichoderma*** ***guizhouense* NJAU4742**

Youzhi Miao, Xing Chen, Tuo Li, Han Zhu, Siyu Tang, Dongyang Liu^*^, Qirong Shen

Jiangsu Provincial Key Lab for Organic Solid Waste Utilization, National Engineering Research Center for Organic-based Fertilizers, Jiangsu Collaborative Innovation Center for Solid Organic Waste Resource Utilization, Nanjing Agricultural University, Nanjing, 210095, P. R. China.

**Running title:** pH-dependent regulation of lignocellulases

*****Address correspondence to Dongyang Liu, [liudongyang@njau.edu.cn](mailto:liudongyang@njau.edu.cn)

Mailing address: College of Resources & Ambient Science, Nanjing Agricultural University, 210095, Nanjing, China, Tel: 86-25-84396477; Fax: 86-25-84396260;

**Additional Figure Captions**

**Table S1. SWATH results of the identified proteins in SSF3.0, SSF6.0 and SSF9.0.**

**Figure S1. Growth conditions of NJAU4742 strain in SSF2.0, SSF3.0, SSF6.0, SSF8.0 and SSF9.0.**

**Figure S2. 2D SDS-PAGE of the total proteins in SSF3.0, SSF6.0 and SSF9.0 for SWATH analysis.**

**Figure S3. Statistical analysis of SWATH results.** After SWATH detection, Frequency distribution of protein coverages and protein abundances were shown in (**A**) and (**B**), respectively. Protein abundance from each repeat was plotted and compared in (**C**).

**Figure S4. Protein expression differences between SSF9.0 and SSF6.0 visualized as a Cytoscape interaction network.**


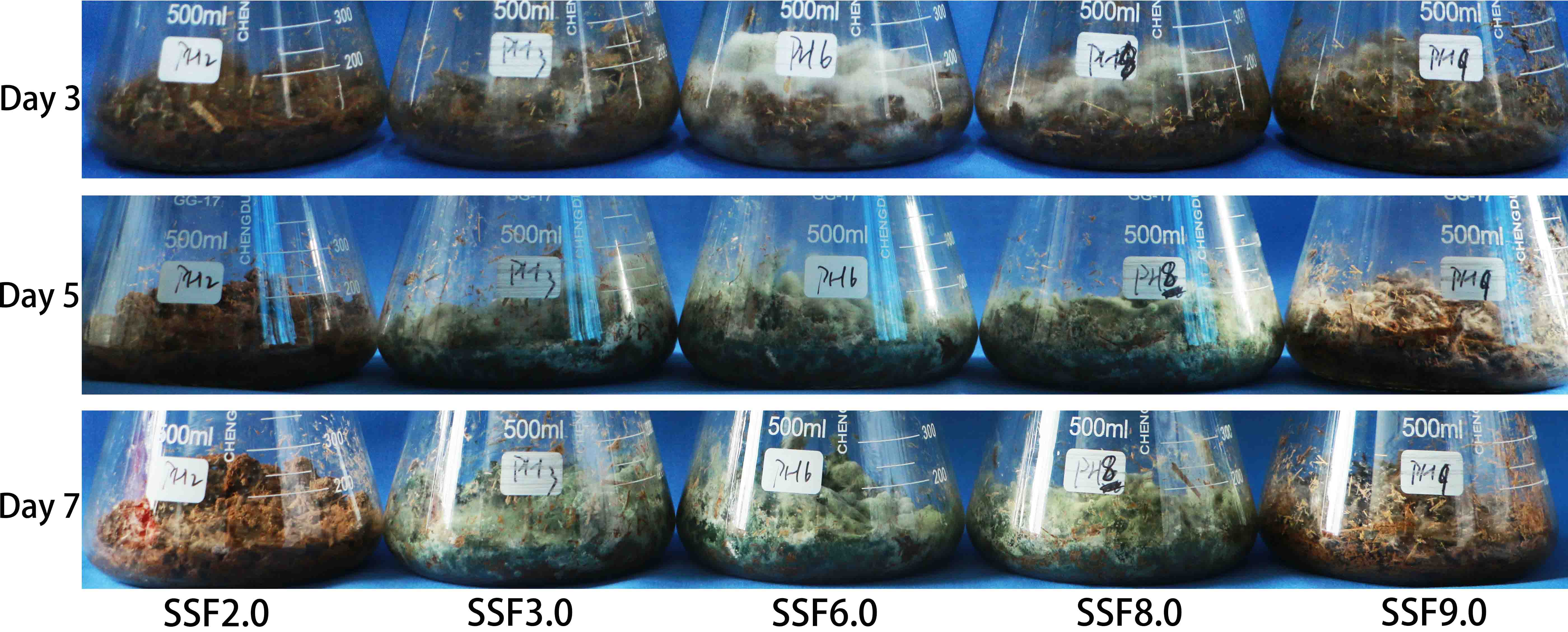


Figure S1


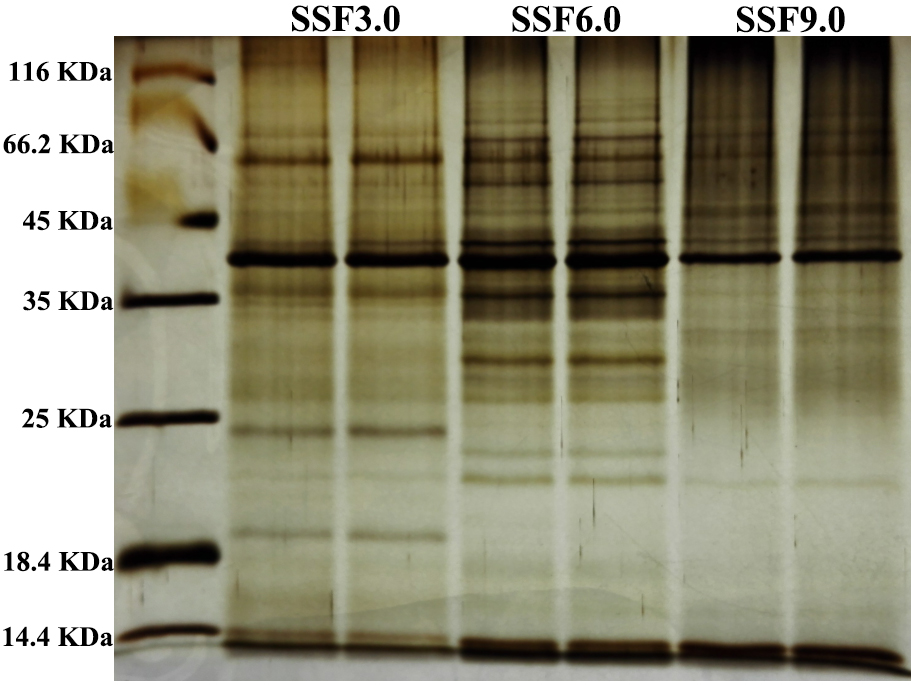


Figure S2


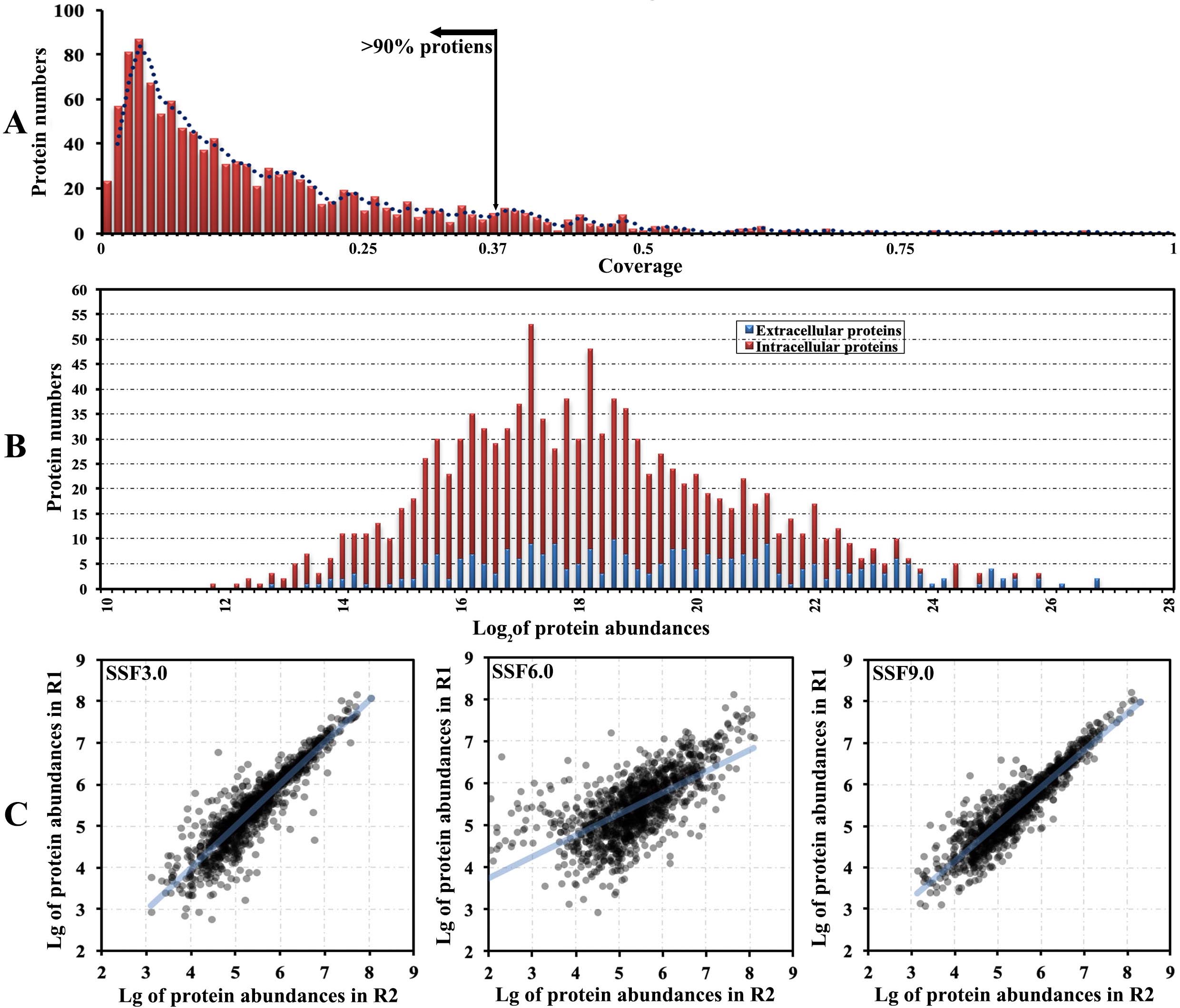


Figure S3
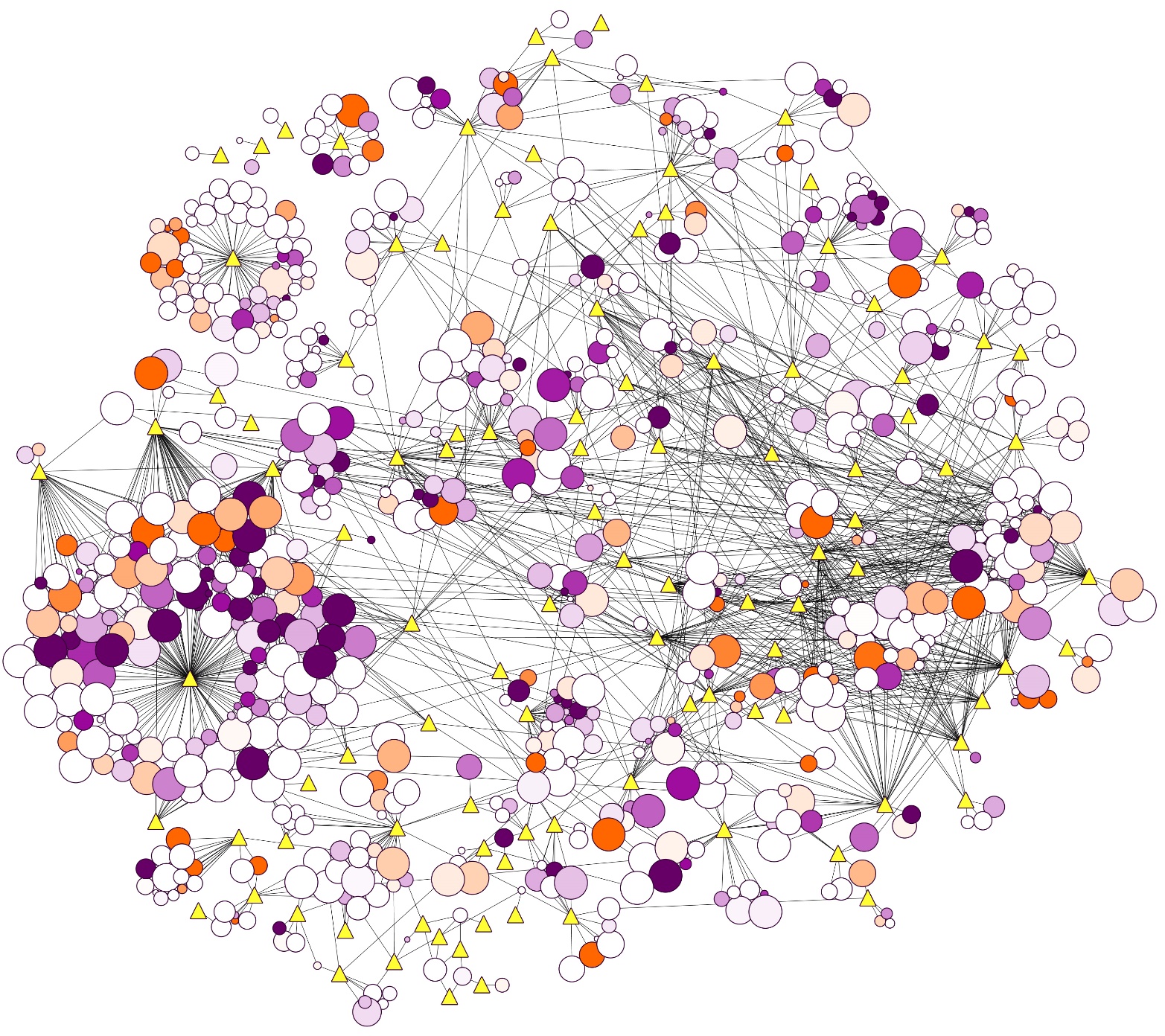


Figure S4
